# Mouse mesenchymal stem cell-derived exosomal miR-466f-3p reverses EMT process through inhibiting AKT/GSK3β pathway via c-MET in radiation-induced lung injury

**DOI:** 10.1186/s13046-022-02351-z

**Published:** 2022-04-07

**Authors:** Yi Li, Zhufu Shen, Xiao Jiang, Yuanyuan Wang, Zuozhang Yang, Yuchi Mao, Zhixian Wu, Gaofeng Li, Hong Chen

**Affiliations:** 1 Department of Oncology, 920th Hospital of Joint Logistics Support Force, Teaching Hospital of Kunming Medical University, 212 Daguan Road, Kunming, 650032 China; 2 Department of Geriatrics, 920th Hospital of Joint Logistics Support Force, Teaching Hospital of Kunming Medical University, Kunming, 650032 China; 3 Department of Pathology, 920th Hospital of Joint Logistics Support Force, Teaching Hospital of Kunming Medical University, Kunming, 650032 China; 4 Department of Orthopaedics, The Third Affiliated Hospital of Kunming Medical University, Kunming, 650118 China; 5 Department of Hepatobiliary Disease, 900th Hospital of Joint Logistics Support Force, Fuzhou, 354200 China; 6 Department of Thoracic Surgery, The Third Affiliated Hospital of Kunming Medical University, Kunming, 650118 China

**Keywords:** Radiation-induced lung fibrosis, Epithelial-mesenchymal transition, Exosome, Mesenchymal stem cells, miR-466f-3p

## Abstract

**Background:**

Radiation-induced lung fibrosis (RILF) is a common complication of thoracic radiotherapy. Alveolar epithelial cells play a crucial role in lung fibrosis via epithelial-mesenchymal transition (EMT). Exosomes derived from mesenchymal stem cells own the beneficial properties to repair and regeneration of damaged tissues, however the underlying mechanisms remain poorly understood.

**Methods:**

Mouse mesenchymal stem cells-derived exosomes (mMSCs-Exo) were isolated by differential centrifugation, and their protective effects were assessed in vivo and in vitro, respectively. EMT-associated proteins were measured via western blot assay and/or immunofluorescence staining. The miRNA expression was measured by microarray assay and qPCR. Furthermore, bioinformatics prediction with KEGG analysis, luciferase assay, and rescue experiments were performed to explore the molecular mechanism underlying miR-466f-3p.

**Results:**

mMSCs-Exos were efficiently isolated ranging from 90-150 nm with high expression of exosomal markers (CD63, TSG101, and CD9). mMSCs-Exos administration efficiently relieved radiation-induced lung injury with less collagen deposition and lower levels of IL-1β and IL-6. Meanwhile, in vitro results showed mMSCs-Exos treatment obviously reversed EMT process induced by radiation. Among enriched miRNA cargo in exosomes, miR-466f-3p was primarily responsible for the protective effects via inhibition of AKT/GSK3β pathway. Our mechanistic study further demonstrated that c-MET was the direct target of miR-466f-3p, whose restoration partially abrogated mMSCs-Exo-mediated inhibition in both EMT process and AKT/GSK3β signaling activity induced by radiation.

**Conclusions:**

Our findings indicated that exosomal miR-466f-3p derived from mMSCs may possess anti-fibrotic properties and prevent radiation-induced EMT through inhibition of AKT/GSK3β via c-MET, providing a promising therapeutic modality for radiation-induced lung fibrosis.

**Supplementary Information:**

The online version contains supplementary material available at 10.1186/s13046-022-02351-z.

## Background

Ionizing radiation (IR) is the most widely used modality for average and advanced lung cancer patients. However, toxic injury in normal lung tissue is a common dose-limiting complication of thoracic radiotherapy, which is categorized into radiation pneumonitis in the early stages and pulmonary fibrosis later on [1]. Radiation-induced lung fibrosis (RILF) remains a lethal disease, and its poor response to current available treatments is still a great obstacle in clinic, which limits the increasing radiation doses and seriously affects patients’ survival and quality of life [2]. Thus, the need for new therapies is paramount.

Epithelial-mesenchymal transition (EMT), a reversible process, has a crucial role in pathogenesis of organ fibrosis [3]. Growing evidences suggest that, after radiation treatment, injured alveolar epithelial cells undergo EMT process, whose acquired mesenchymal-like properties are essential for progressive accumulation of collagen and extracellular matrix proteins, ultimately leading to lung fibrosis [4, 5]. Besides transforming growth factor-β (TGF-β), multiple signaling pathways have also been appeared in radiation-induced lung EMT [6, 7]. Previous studies indicate that AKT signaling in normal alveolar epithelial cells is necessary for regulation of radiation-induced EMT [8]. AKT is a key component in numerous processes, which could induce EMT through suppression of E-cadherin based on major transcription factors such as Snail and Twist [9], suggesting that AKT signaling pathway could be employed as a potential target to attenuate radiation-induced EMT [10]. Furthermore, the exact mechanism are not fully elucidated.

Exosomes are double-layered lipid membrane vesicles that are secreted by different cell types [11]. Growing evidence suggests that exosomes owning the beneficial properties of their parental cells are important mediators of intercellular communication via loading a complex cargo such as miRNAs, proteins, and lipids [12]. Although mesenchymal stem cells (MSCs) have shown the promise for tissue repair in several models of clinic and experimental diseases characterized with low level of fibrosis [13, 14], there is a growing awareness that their efficiency is mainly attributed to the secretion of soluble paracrine factors and extracellular vesicles, especially exosomes [15, 16]. A recent study showed that MSCs-derived exosomes enriched with miRNA-182-5p and miR-23a-3p benefited LPS-induced acute lung injury through the inhibition of EMT. Increased miR-214-3p derived from MSCs-derived extracellular vesicles contributed to attenuation of the radiation-induced injury of endothelial cells [17]. Given that miRNAs are key regulators of lung fibrosis, with the benefit of easy detection and well stability, it is worthwhile to unveil the miRNA profile released by MSCs-Exo in treating radiation-induced lung fibrosis.

In this study, we focused on the *in-vitro* and *in-vivo* protective role of mMSCs-derived exosomes in radiation-induced lung injury (RILI). Then, the underlying mechanism was explored via miRNAs microarray, and the enriched miR-466f-3p was identified in mMSCs-Exos. More importantly, we demonstrated that miR-466f-3p overexpression can alleviate radiation-induced EMT in alveolar epithelial cells through AKT/GSK3β signaling pathway via targeting c-MET, supporting the potential utility of exosomal miR-466f-3p for RILI treatment.

## Methods and Materials

### Isolation and characterization of mouse MSCs from bone marrow and cell culture

Mouse mesenchymal stem cells (mMSCs) was isolated from bone marrow and cultured in DMED medium (Hyclone) supplemented with 10% exosome-free fetal bovine serum (FBS; Gibco) as previously described [18]. The cells were passaged with 0.25% trypsin containing 0.02% ethylenediaminetetraacetic acid until they reached 90% confluency. A purified population of mMSCs with spindle-shaped morphology was obtained 3 weeks after initial culture. To analyze the multipotential, mMSCs were cultured in appropriate induction medium, and the osteogenic, adipogenic, and chondrogenic differentiation was stained in Alizarin Red, Oil Red, and Alcian Blue, respectively, according to the manufacturers’ instruction. Flow cytometry was used to analyze the phenotypic characteristics of mMSCs. The cells were stained with mouse conjugated monoclonal antibodies, including CD44-APC (103012, BioLegend, USA), CD34-PE (128609, BioLegend, USA), CD11b-PE (101207, BioLegend, USA), CD90.2-PE (105311, BioLegend, USA), and Sca-1-PE (108107, BioLegend, USA). And identical concentrations of the corresponding IgG isotype antibodies were used as negative control (BD biosciences, CA). A minimum of 10000 events were acquired on a FACS instrument (BD bioscience, CA). FlowJo software was employed to analyze the results. The mouse alveolar epithelial cells MLE-12 were purchased from ATCC (Manassas, VA) and routinely maintained in RPMI 1640 medium (Hyclone, UT) with 10% exosome-free FBS and 1% pen/strep (Gibco). Cells were routinely maintained in a humidified incubator containing 5% CO_2_ at 37 °C. Exosome-free PBS were prepared by pelleting the exosomes by ultracentrifugation at 100000*g* for 3 h at 4 °C, and the resulting supernatant was filtered through a 0.2-μm filter.

### Isolation and characterization of exosomes

mMSCs were cultured with 10% exosome-free FBS, and cell culture medium were harvested every 2 days, and differentially centrifuged at 300*g* for 10 min, 2000*g* for 15 min and 10000*g* for 30 min to remove floating cells and cellular debris. Then the supernatants were filtered through a 0.22-μm filter and ultracentrifuged again at 100000*g* at 4 °C for 1 h (Beckman Coulter, Inc., USA). The exosome-enriched pellet was washed with 10 ml of 1 × PBS and pelleted again by ultracentrifugation at 100000*g* at 4 °C for 1 h. The resulting pellet was suspended in 1 × PBS for whole exosome application. The size distribution of exosomes was measured by nanoparticle tracking analysis (NTA) according to the operating instructions (Nano Sight NS300, Malvern, United Kingdom). The exosomal surface markers CD63, TSG101, and CD9 (#EXOAB series, System Biosciences), as well as the negative marker GM130 (ab187514, Abcam, USA), were detected via western blot assay. Additionally, exosome morphology was visualized by transmission electron microscopy (Hitachi, Japan) as previously described.

### Cell radiation and treatment

MLE-12 cells were seeded into the 6-well plates. When MLE-12 cells grew at about 70% confluency, a total of 40 μg mMSCs exosomes were added into each well directly, and PBS was used as control. 24 h later, MLE-12 cells with or without exosomes treatment were subjected to a single dose of 8 Gy radiation via a 300 KV X-ray machine (HITACHI, Japan) at the room temperature. Then the cells were harvested for the following experiments at 48 h post-irradiation. To block the exosome generation, GW4869 (Sigma, USA) was added into mMSCs for 48 h at a final concentration of 5μM. Next, MLE-12 cells were incubated with the above medium one day before radiation exposure, and followed by collection as mentioned above.

### Mouse model with radiation-induced lung injury and treatments

Seven-week-old C57BL/6 mice were maintained under standard laboratory conditions for 1 week prior to treatment. Mice were divided into two groups (16 mice/group) and anesthetized with 10% choral hydrate before their whole thorax exposed to a single dose of 14 Gy radiation. The treated animals received a i.v. (tail vein) dose of exosomes (200 μg per mouse suspended in 400 μl PBS) 2 h before radiation, then repeated once two weeks until sacrifice. Exosomes naïve mice (control group) only received PBS via the same route. Mice were humanely sacrificed at 1 w, 4 w, 8 w, and 12 w to collect serum and lung samples for further experiments. Efforts were made to ensure the animals suffered minimally. All animal experiments were approved by the Institutional Animal Experiment Committee at 920^th^ Hospital.

### Histology, Immunohistochemistry and Masson’s trichrome staining

The left lung tissue in each mouse was fixed in 10% formalin for at least 48 h. After which, they were dehydrated with a series of acetone, and embedded in paraffin. The samples were sliced into 4-μm slices and stained with hematoxylin and eosin (H&E), or using a Masson’s trichrome Kit (Sigma-Aldrich, USA) to detect collagen. For immunohistochemistry (IHC) staining, samples were blocked with 3% hydrogen peroxide for 30 min and then incubated at 4 °C overnight with primary antibodies against E-cadherin (#3195, cell signal technology, USA), Vimentin (#5741, cell signal technology, USA) at a dilution of 1:100. After washing, the sections were incubated at 37 °C with appropriate biotinylated secondary antibody (Zhongshan, China). The sections were observed with the light microscope (Olympus, Japan), and the protein expression was analyzed as previously described [19].

### Hydroxyproline assay

The collagen concentration was measured by Hydroxyproline (Hyp) assay Kit (Solarbio life science, China) according to the manufacturer’s protocol. Some lung tissue samples were hydrolyzed in lysis buffer solution at 110 °C for 2h. Absorbance of colored products was measured at 560 nm. The Hyp content was calculated as micrograms of Hyp per milligram of wet weight (μg/mg).

### Enzyme-linked immunosorbent (ELISA) assay

At each time-point post-irradiation, cardiac puncture was performed to obtain the whole blood. The blood was allowed to clot at room temperature for 2 h and centrifuged at 3000 g 4 °C for 15 min. The clear top layer was collected, and the levels of interleukin-1β (IL-1β, ab197742, Abcam, USA) and interleukin-6 (IL-6, ab222503, Abcam, USA) were measured using a commercial ELISA kit following the manufacturer’s protocol. The OD value of each well was measured at 450 nm via a Microplate Reader (Thermo Fisher Scientific Inc., USA) and calculated at the linear portion of the curve.

### Exosomes labeling and confocal microscopy

Exosomes isolated from mMSCs were pre-treated with the green lipophilic fluorescent dye PKH67 according to the instructions (Sigma-Aldrich, USA). Subsequently, the fluorescently labelled exosomes with a final concentration of 10 μg/mL were added into MLE-12 cells. After incubation for the indicated time points, MLE-12 cells were fixed with 4% paraformaldehyde for 30 min at room temperature. Then the cells were stained with mounting medium containing 4’, 6-diamidino-2-phenylindole (DAPI; Invitrogen, USA) for 5 min. After staining, cells were visualized under confocal laser-scanning microscopy (Olympus, Japan).

### Cell transfection

Mature mmu-miR-466f-3p mimic, mmu-miR-466f-3p inhibitor, or mmu-antagomir-466f-3p (for *in-vivo* assay), and individual scrambles were purchased from RuiBoBio Company (Guangzhou, China). The CDS sequence of c-Met without 3’UTR was cloned into the pcDNA-3.0 plasmid to construct the pcDNA-c-Met vector. And siRNA against c-MET (Invitrogen) was used to knockdown c-MET expression. When cells reached 50% confluency, miRNA mimics (50 nM) or inhibitor (100nM) was transfected with Lipofectamine 3000 Kit (Invitrogen, USA) and Opti-MEM serum-free medium (Invitrogen, USA) according to the manufacturer’s instructions. 48 h after transfection, cells or conditional medium were harvested for the following experiments.

### Transmission electron microscopy (TEM)

Cells or exosomes were fixed with a solution containing 3% glutaraldehyde plus 2% paraformaldehyde in 0.1% mol/l phosphate buffer, followed by 1% OSO4 overnight, dehydrated with a series of acetone, embedded and solidified. Then, the samples were sliced into 50-nm ultrathin sections to observe the intracellular structures via TEM HT7700 (Hitachi, Japan) as described previously [20].

### Microarray analysis of miRNAs

The miRNA profile between irradiated MLE-12 cells with or without mMSCs-Exos treatment was performed at KangCheng Biotechnology Corporation (Shanghai, China). Agilent Mouse miRNA microarray (Agilent Technologies, USA) was used in the analysis. According to the manufacturer’s protocol, miRNAs were labeled and hybridized with miRNA complete Labeling and Hybridization kit. Data normalization and processing were performed using Quantile algorithm, Gene Spring Software 12.6 (Agilent Technologies, USA). The differential expression of miRNAs was performed via the Pearson’s correlation analysis with Cluster 3.0 and TreeView software, and the differentially expressed genes (DEGs) were identified to have at least |logFC| > 2, *p* value < 5% in expression.

### Quantitative RT- PCR

According to the manufacturer’s protocol, total RNA was extracted from cells and exosomes via TRIZOL and TRIZOL LS reagent, respectively (Invitrogen, USA). Complementary DNA (cDNA) was synthesized using the Reverse Transcription Kit (Takara, Japan) under the following conditions:42 °C for 1 h and then 95 °C for 5 min. Prior to isolation of exosome RNA, *C. elegans* cel-miR-39 (0.25 nM) standard RNA (RiboBio, China) was added to each sample as a spike-in control. PCR reactions were performed in triplicate on the ABI Prism 7900 (Applied Biosystems, USA) using the SYBR PCR Master Mix (Takara, Japan). The sequences of primers were presented in Table S1. The relative expression of individual genes was analyzed by the 2^-△△Ct^ method through normalizing to U6 or cel-miR-39.

### Western blot assay

Protein was extracted from cells by a protein extraction reagent (Roche, Switzerland), and measured by a BCA protein kit (Pierce Biotechnology, USA). 40-μg protein for each sample was electrophoresed in 10-15% SDS gel, then transferred to nitrocellulose membranes. After blocked with 5% BSA solution at room temperature for 1 h, membranes were incubated with primary antibodies at a dilution of 1/1000 at 4 °C overnight, including E-cadherin (#3195, cell signal technology, USA), Vimentin (#5741, cell signal technology, USA), Snail (#3879, cell signal technology, USA), c-MET (ab216330, Abcam, USA), total AKT (ab38449, Abcam, USA), p-AKT (ab8805, Abcam, USA), GSK3β (ab227208, Abcam, USA), p-GSK3β (#9322, cell signal technology, USA), or GAPDH (ab181602, Abcam, USA). Then, followed with secondary antibody (1:5000) at 37°C for 1h. The signals were detected by ECL Kit (Pierce Biotechnology, USA), and were analyzed by Image pro plus software.

### Immunofluorescence staining

For immunofluorescence staining, cells cultured in six-well plates were fixed in 4% paraformaldehyde for 20 min, washed with PBS, and next permeabilized with 0.1% Triton X-100 for 30 min at room temperature. Then cells were blocked with 5% bovine serum albumin for 1 h, and incubated with primary antibodies against E-cadherin (#3195, cell signal technology, USA), Vimentin (#5741, cell signal technology, USA), and Snail (Bioss, bs-1371R, CHINA) at dilutions of 1:200 at 4 °C overnight. After washing, the sections were incubated with fluorescent secondary antibodies at room temperature for 30 min. Nuclei were counterstained with DAPI, and the slides were observed via a confocal laser-scanning microscope (Olympus, Japan).

### Luciferase reporter assay

The predicted miR-466f-3p-binding sequences in c-Met 3’-UTR was amplified by PCR and inserted into pmirGLO vector (Promega, USA) to construct luciferase reporter vector (pmirGLO-c-Met-wt). Similarly, the potential binding sites of miR-466f-3p in the above sequences were mutated by Quickchange Mutagenesis Kit (Agilent Technologies, USA) to construct mutant vectors, labeled as pmirGLO-c-Met-mut. H293T cells were seeded into 12-well plates at a density of 1×10^5^ cells/well. 24 h later, miR-466f-3p mimics or scrambles were co-transfected with recombinant wide-type or mutant vectors by Lipofectamine 3000 (Invitrogen, USA). The empty pmirGLO vector was transfected as control. The luciferase activities were standardized to the value of the co-transfected group with an empty vector and scrambles.

### Statistical analysis

The data were presented as means ± standard deviation (SD.) of three independent experiments. All statistical analysis were performed by GraphPad Prism software. The differences among control and experimental groups were measured by Two-tailed student’s *t*-tests, whereas ANOVA calculated differences among multiple groups. *P*-value < 0.05 was considered to be statistically significant.

## Results

### Characteristics of mouse mesenchymal stem cells and their exosomes

**Fig. 1:**
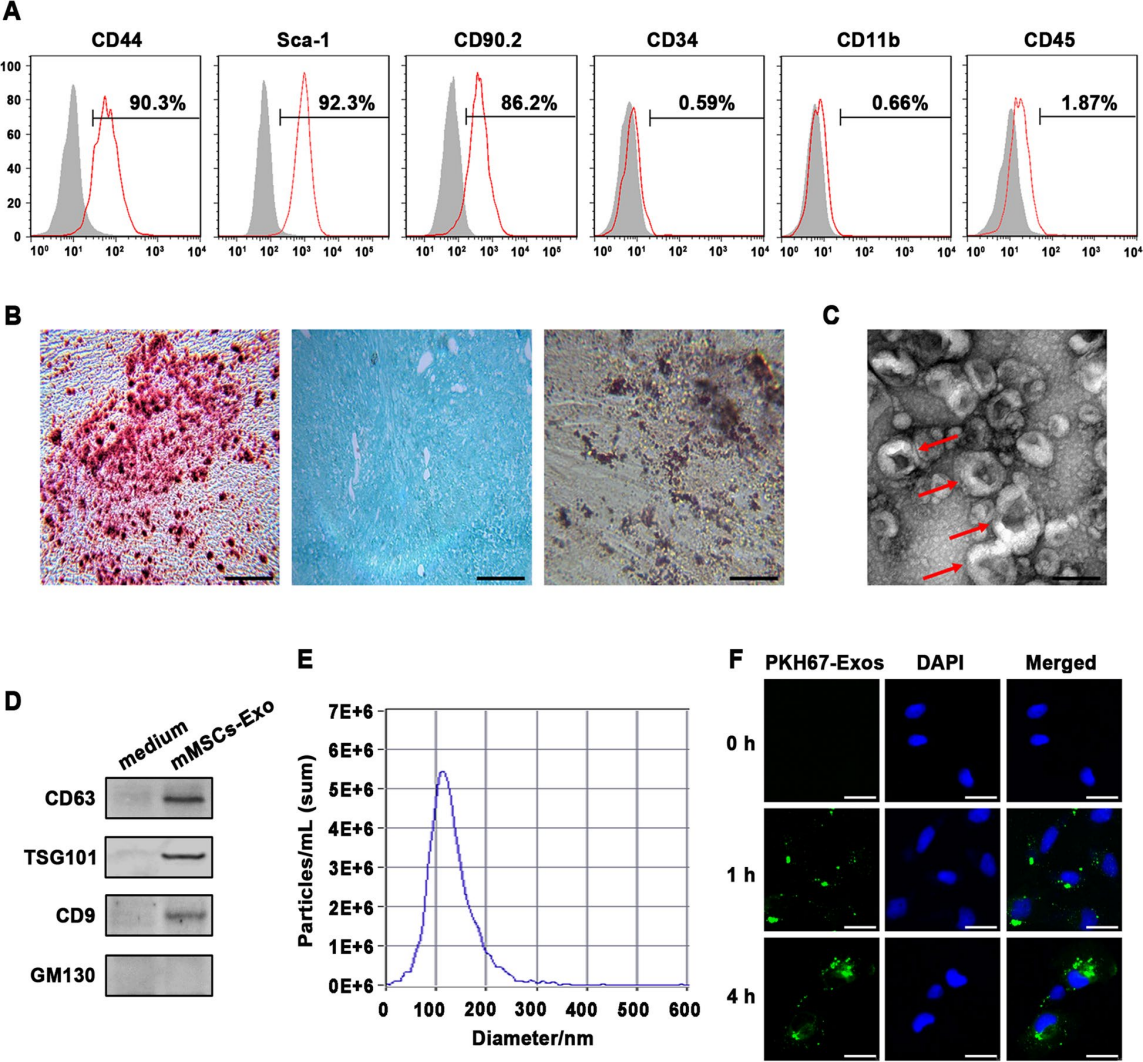
Identification of mouse mesenchymal stem cells (mMSCs) and mMSCs-derived exosomes. **A** Flow cytometric analysis for cell surface markers of CD44, Sca-1, CD90.2, CD34, CD11b, and CD45 on mMSCs. Percentage of positive cells shown in upper right corner as standardized with the isotype control antibody-incubated cells. **B** Assessment of differentiating capacity of mMSCs into osteocytes, chondrocytes, and adipocytes stained by Alizarin Red *(left)*, Alcian Blue *(middle)*, and Oil Red *(right)*, respectively. Scale bar = 100 μm. **C** Representative image of transmission electron microscopy of mMSCs-Exo, and red arrows indicate typical exosomes. Scale bar = 200 nm. **D **Western Blot analysis for exosomal markers CD63, TSG101, and CD9, as well as the negative marker GM130 in mMSCs-Exo. The culture medium was used as control. **E **Nanoparticle tracking analysis of mMSCs-Exo.** F** Confocal images shown that PKH67-labeled exosomes (green) were taken up by MLE-12 cells at indicated time points in vitro. Nuclei were counterstained with DAPI. Scale bar = 40 μm

After 3-weeks culture, a population of mouse mesenchymal stem cells (mMSCs) purified from bone marrow were analyzed via flow cytometry assay, which positively expressed the typical MSC markers CD44, CD90.2, and Sca-1. Whereas the hematopoietic markers, such as CD34, CD11b and CD45, were negative (Fig. 1A). Upon appropriate induction medium, the isolated mMSCs showed their differentiation capacities into osteocytes, adipocytes, and chondrocytes, which positively stained with Alizarin Red, Oil Red, and Alcian Blue, respectively (Fig. 1B). Then, exosomes were isolated from the conditional medium of mMSCs via standard ultracentrifugation and identified using TEM, Nanoparticle tracking analysis, and Western blot assay, respectively. As shown in Fig. 1C-E, the typical exosomes exhibited round-shaped morphology, ranged from 90 to 150 nm in size, and positively expressed the common exosomal markers, including CD63, TSG101 and CD9. While intracellular protein GM130 was absent in the exosomes. Afterward, to evaluate whether exosomes derived from mMSCs were absorbed by recipient cells, PKH67-labelled mMSCs-Exos were co-cultured with murine alveolar epithelial cells MLE-12, which were chosen for study due to their crucial function in RILI. And we visualized that the PKH67 signal was increasingly accumulated in the cytoplasm of MLE-12 in a time-dependent manner, reaching a maximum after 4 h of incubation via confocal microscopy (Fig. 1F).

### mMSCs-Exos prevent radiation-induced alveolar EMT in vitro

**Fig. 2:**
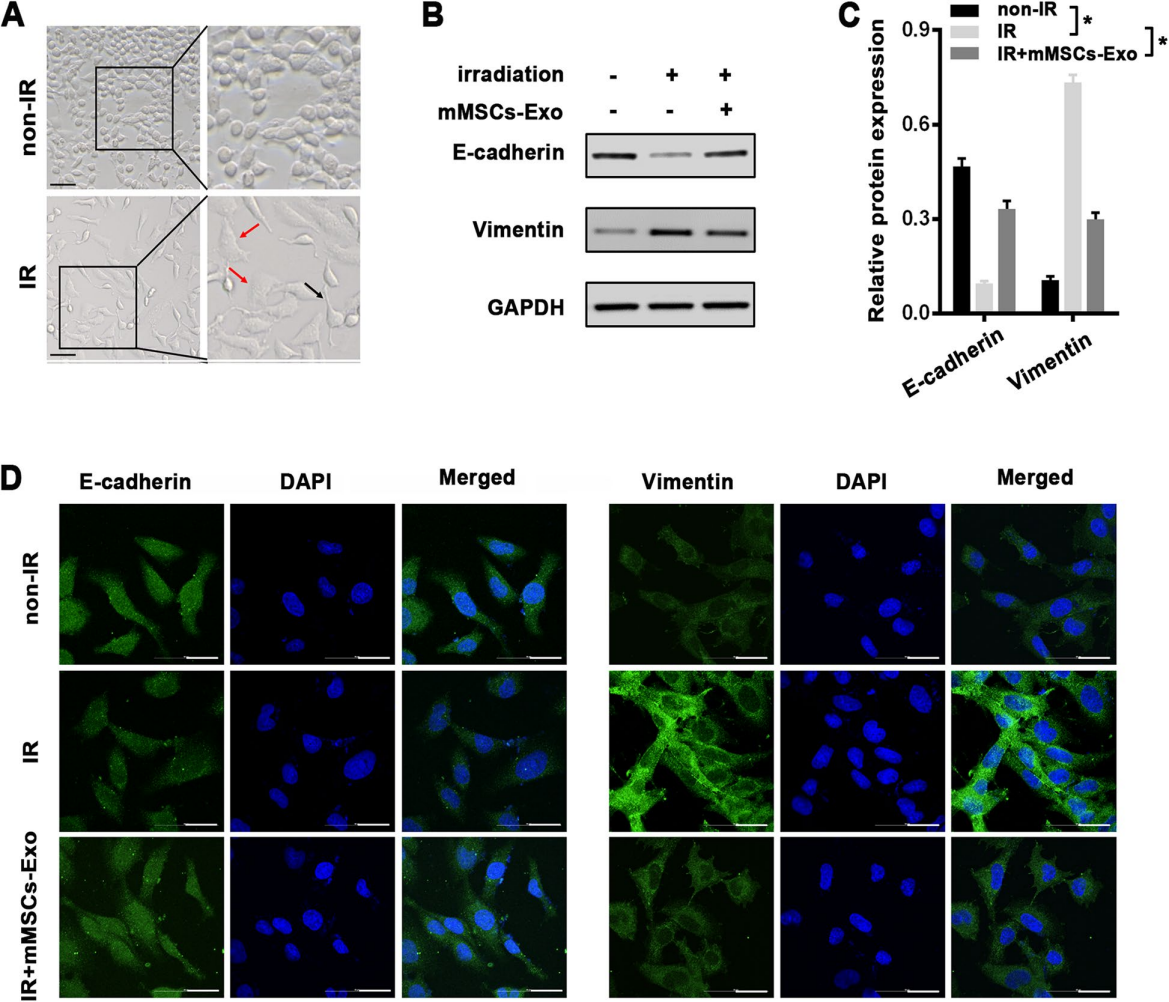
mMSCs-Exo attenuated radiation-induced EMT in MLE-12 cells. **A** Representative images of cell morphology at 48 h after a single dose of 8 Gy radiation or non-radiation. Zoom-in images were shown to closely observe the epithelial or mesenchymal morphology. Arrows represent extended pseudopodia (black) and elongated appearance (red). Scale bars = 100 μm. **B**, **C** Western Blot analysis (B) and densitometric quantification (C) of E-cadherin, Vimentin and GAPDH (for normalization) in nonirradiated control and irradiated cells with or without mMSCs-Exo. **D** Immunofluorescence staining for E-cadherin, Vimentin (green), and DAPI (blue) in nonirradiated control and irradiated cells with or without mMSCs-Exo. Scale bars = 100 μm. Data are presented as the mean ± SD. from three independent experiments, * *p* < 0.05

In response to radiation, alveolar epithelial cells underwent EMT process, which has been identified as a significant mechanism for the pathogenesis of RILI [21]. As expected, MLE-12 cells irradiated with a single dose of 8 Gy presented an obvious morphological changes, and most of the cells transformed from a typical epithelium into an elongated mesenchymal-like appearance with extended pseudopodia at 48 h post-irradiation (Fig. 2A). To explore the roles of mMSCs-Exos in radiation-induced EMT, MLE-12 cells was pretreated with mMSCs-Exos for 4 h prior to radiation, and then EMT-associated markers were analyzed by western blot after another 48 h. In line with radiation-induced morphological changes, the EMT statue in irradiated MLE-12 cells was confirmed by an obvious decrease of epithelial marker (E-cadherin) and an enhanced expression of mesenchymal marker (Vimentin) as compared to the nonirradiated control cells (Fig. 2B, C). While pretreatment of mMSCs-Exos obviously reversed the radiation-induced EMT, with higher E-cadherin and lower Vimentin expression than those in radiation-only cells (Fig. 2B, C). Moreover, the above changes of EMT-associated proteins were further identified by immunofluorescence (Fig. 2D).

### mMSCs-Exos prevent radiation-induced lung injury in vivo

**Fig. 3:**
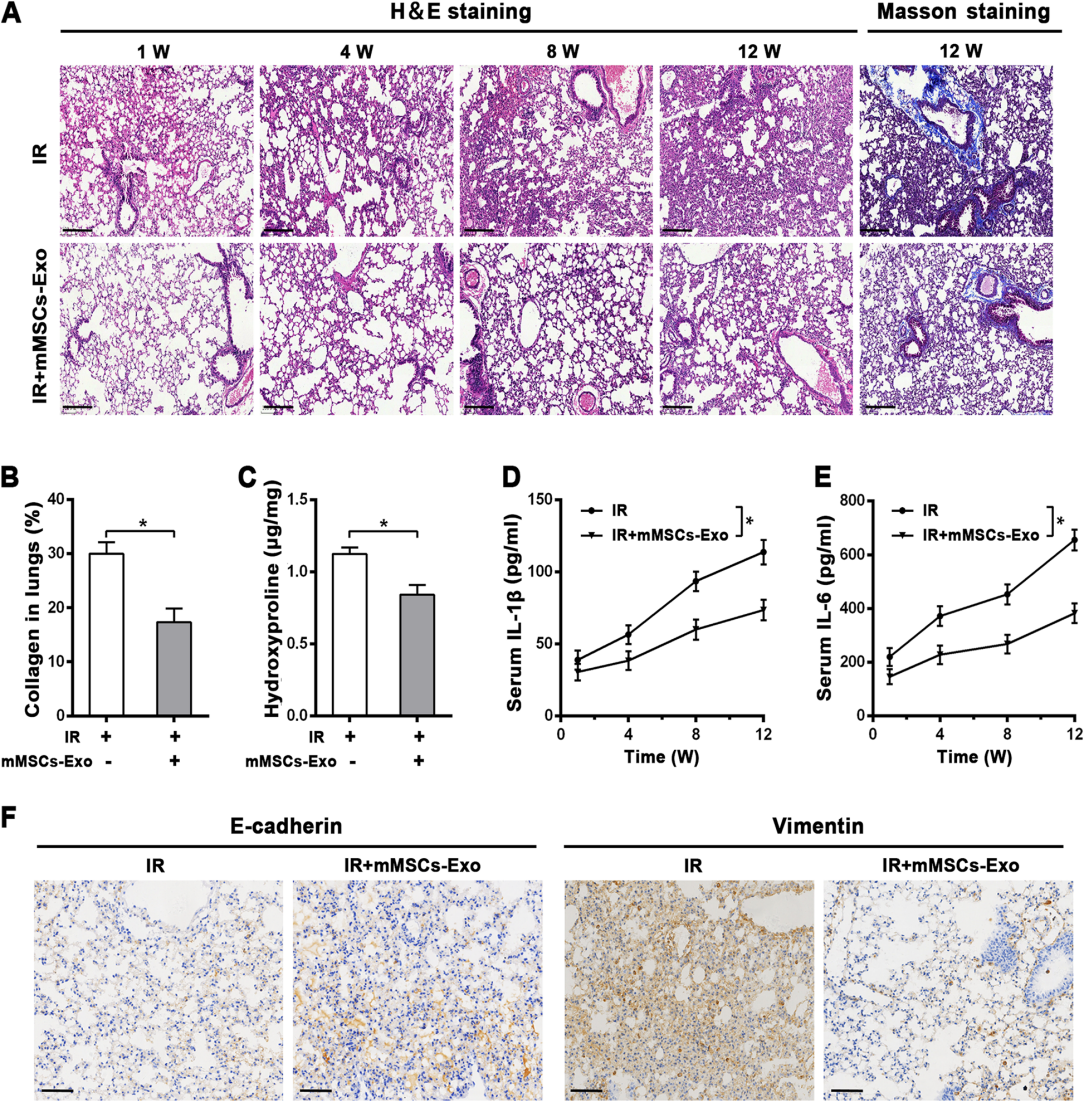
mMSCs-Exo alleviated radiation-induced lung injury (RILI) and fibrosis in vivo. **A** H&E and Masson staining in lung tissues from the mouse model of RILI with or without mMSCs-Exo at the indicated time points post-irradiation, *n* = 4 mice per group. Representative images were presented. Scale bars = 200 μm. **B** Percentage of collagen deposition in lung sections was quantified from different groups at 12 w post-irradiation. **C** Biochemical assay for Hydroxyproline content in lung sections from different groups at 12 w post-irradiation, shown as microgram of Hyp per milligram of wet weight (μg/mg). **D**, **E** ELISA assay for proinflammatory cytokine IL-1β (D) and IL-6 (E) in serum from different groups at the indicated time points post-irradiation. **F** Representative images of IHC assay for E-cadherin and Vimentin proteins from different groups at 12 weeks post-irradiation, Scale bars = 100 μm. Data are presented as the mean ± SD. from three independent experiments, * *p* < 0.05

To investigate the cytoprotective role *in-vivo*, a mouse model of RILI was established via a single dose of 14 Gy to the whole thorax. For the treated group, the mice were intravenously injected with mMSCs-Exos 2 h before radiation. Interestingly, as shown via H&E staining, administration of mMSCs-Exos obviously attenuated radiation-induced lung injury at 1 week, 4 weeks, 8 weeks, and 12 weeks, in which the pathological damages, including thickened alveolar septa, interstitial oedema, and infiltrated inflammatory cell, were obviously mitigated. And lung alveolar integrity was better than that in radiation only group (Fig. 3A). Notably, Masson staining further revealed that collagen was extensively deposited at 12 weeks post-irradiation, particularly around blood vessels, whereas the deposition was reduced by mMSCs-Exos (Fig. 3A, B). Besides, the hydroxyproline content, a reliable marker of collagen protein, was measured via a biochemical assay. In line with Masson’s staining, mMSCs-Exos also blunted the radiation-induced increase of hydroxyproline content in lung tissues at 12 weeks after radiation (Fig. 3C). Additionally, we performed ELISA assay to measure the inflammatory cytokines, which may contribute to the development of fibrosis via promoting the maturation of fibroblasts and excess deposition of extracellular matrix [22]. The data showed that, while the secretion and release of IL-1β and IL-6 were markedly increased at 12 weeks post-irradiation, mMSCs-Exos administration significantly inhibited their levels when compared with radiation-only group (Fig. 3D, E). Furthermore, IHC data revealed that, mMSCs-Exos led to obviously vary in the EMT-associated proteins at 12 weeks post-irradiation, with an increased expression of E-cadherin and a decrease in Vimentin as compared to radiation-only group (Fig. 3F).

### Exosomal mmu-miR-466f-3p from mMSCs reverses EMT and lung injury induced by radiation in vitro and in vivo

**Fig. 4:**
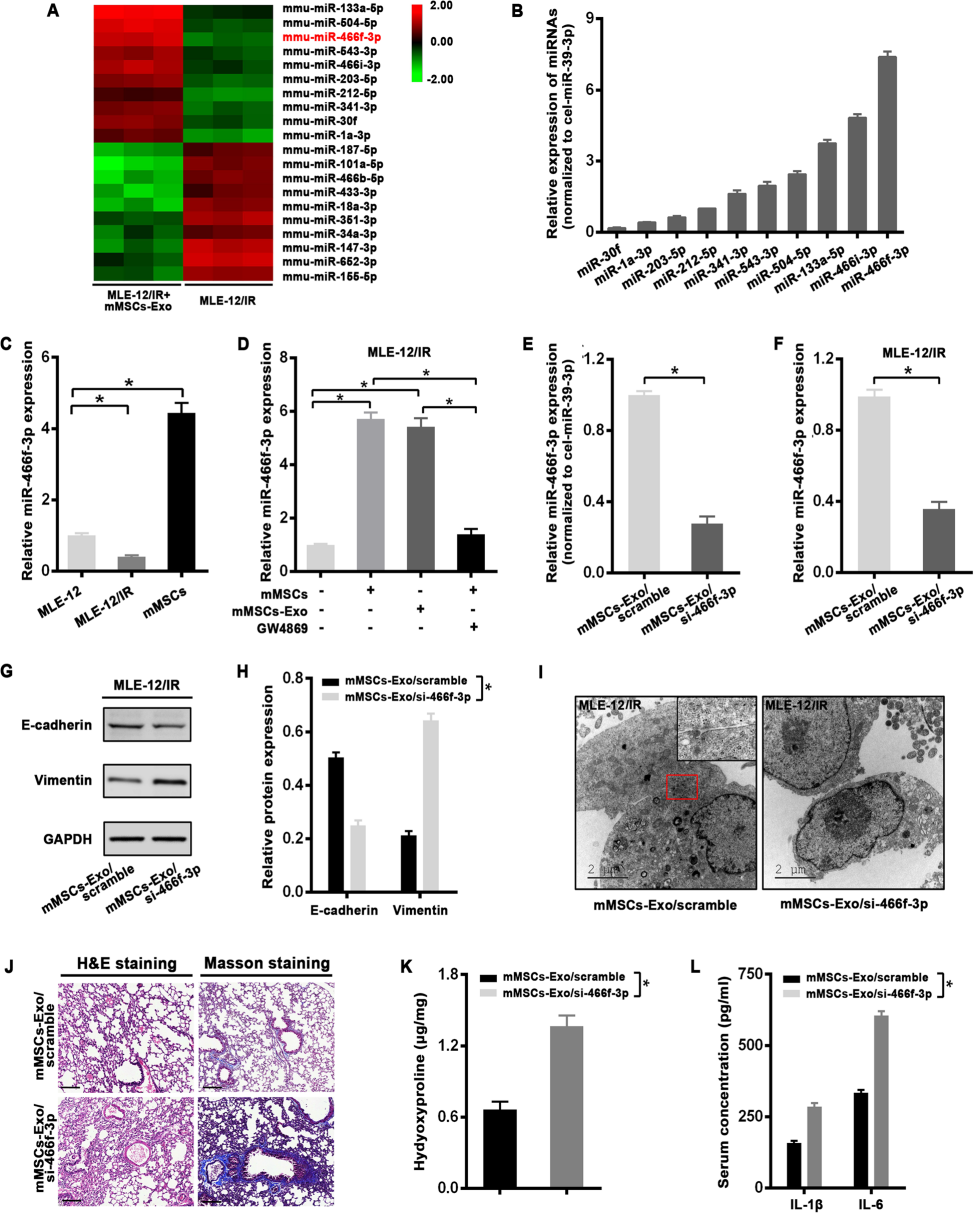
Treatment with mMSCs-Exo reversed radiation-induced EMT in MLE-12 cells via miR-466f-3p. **A** MiRNA microassay data of irradiated MLE-12 cells pretreated with or without mMSCs-Exo were shown in the Heatmap. **B** qRT-PCR analysis of miRNAs expression in mMSCs-Exo. Cel-miR-39-3p was used as an internal control. **C** qRT-PCR analysis of miR-466f-3p expression in MLE-12 cells with or without radiation and mMSCs cells. U6 was used as an internal control. **D** qRT-PCR analysis of miR-466f-3p expression in irradiated MLE-12 cells cocultured with mMSCs, mMSCs-Exo, or GW4869. U6 was used as an internal control. **E** qRT-PCR analysis of miR-466f-3p in scramble and miR-466f-3p-inhibiting exosomes. Cel-miR-39-3p was used as an internal control. **F** qRT-PCR analysis of miR-466f-3p in irradiated MLE-12 cells pretreated with scramble or miR-466f-3p-inhibiting exosomes (mMSCs-Exo/si-466f-3p). U6 was used as an internal control. **G**, **H** Western Blot analysis (G) and densitometric quantification (H) of E-cadherin, Vimentin, and GAPDH (for normalization) in irradiated MLE-12 cells pretreated with scramble or miR-466f-3p-inhibiting exosomes. **I** Representative images of transmission electron microscopy for irradiated MLE-12 cells pretreated with scramble or mMSCs-Exo/si-466f-3p. Zoom-in images were shown to closely observe cellular junctions. Scale bars = 2 μm. MLE-12/IR: MLE-12 cells exposed to 8 Gy radiation. **J** H&E and Masson staining in lung tissues from the mouse model of RILI treated with scramble or mMSCs-Exo/si-466f-3p at 8 w post-irradiation, n = 4 mice per group. Scale bars = 200 μm. **K** Biochemical assay for Hydroxyproline content in lung sections from different groups at 8 w post-irradiation, shown as microgram of Hyp per milligram of wet weight (μg/mg). **L** ELISA assay for proinflammatory cytokine IL-1β and IL-6 in serum from different groups at 8 w post-irradiation. Data are presented as the mean ± SD. from three independent experiments, * *p* < 0.05

Given that the shuttle of miRNAs has been considered as an important functional role for exosomes, we conducted miRNA microarray to screen the profile required for the protective effect against IR. In particular, a substantial increase in miRNAs was observed in irradiated MLE-12 cells cocultured with mMSC-Exos as compared to radiation-only cells (Fig. 4A), than their expression was validated by qRT-PCR. Among the top 10 upregulated miRNAs, mmu-miR-466f-3p (miR-466f-3p) was most enriched in mMSCs-Exos (Fig. 4B). The level of miR-466f-3p was higher in mMSCs than that in MLE-12 cells, which was significantly downregulated in MLE-12 cells after radiation exposure (Fig. 4C). Specially, treatment with either mMSCs or mMSCs-Exos significantly increased miR-466f-3p expression in irradiated MLE-12 cells. However, the trend was impaired by the exosome inhibitor GW4869 (Fig. 4D). Furthermore, to elucidate the role of exosomal miR-466f-3p in radiation-induced EMT, miR-466f-3p inhibitor was introduced into mMSCs, which marked decreased miR-466f-3p expression in mMSCs-derived exosomes (mMSCs-exo/si-466f-3p) as compared to exosomes from scramble-transfected mMSCs (mMSCs-exo/scramble) (Fig. 4E). As expected, the level of miR-466f-3p in irradiated MLE-12 cells with scrambled exosomes was higher than that in mMSCs-exo/si-466f-3p-treated cells (Fig. 4F). Interestingly, treatment with miR-466f-3p-inhibiting exosomes (mMSCs-exo/si-466f-3p) significantly reduced E-cadherin expression and elevated the Vimentin when compared to irradiated MLE-12 cells with scrambled control (Fig. 4G, H). Additionally, as shown by transmission electron microscopy (TEM), the irradiated cells co-incubated with scrambled exosome still maintained some epithelial properties, such as adhesion junctions, whereas treatment with miR-466f-3p-inhibiting exosomes kept the cells in a dispersed pattern with no functional junctions (Fig. 4I).

Subsequently, we examined the preventive role of exosomal miR-466f-3p in a mouse model with RILI. At 8 weeks post-irradiation, H&E and Masson staining revealed that there were more serious pathologic pulmonary damage, as well as more collagen deposition in the mice with miR-466f-3p-inhibiting exosomes, whereas the above-mentioned injury was obviously relieved by scrambled control (Fig. 4J). Hydroxyproline assessment presented that scrambled exosomes induced a marked decrease in the lung tissue at 8 weeks after radiation, whereas the content was significantly increased in the lungs of mice with miR-466f-3p-inhibiting exosomes treatment (Fig. 4K). Furthermore, in comparison to the scrambled exosome, miR-466f-3p inhibition also resulted in a higher levels of IL-1β and IL-6 (Fig. 4L).

### miR-466f-3p-dependent AKT/GSK3β pathway is critical for radiation-induced EMT

**Fig. 5:**
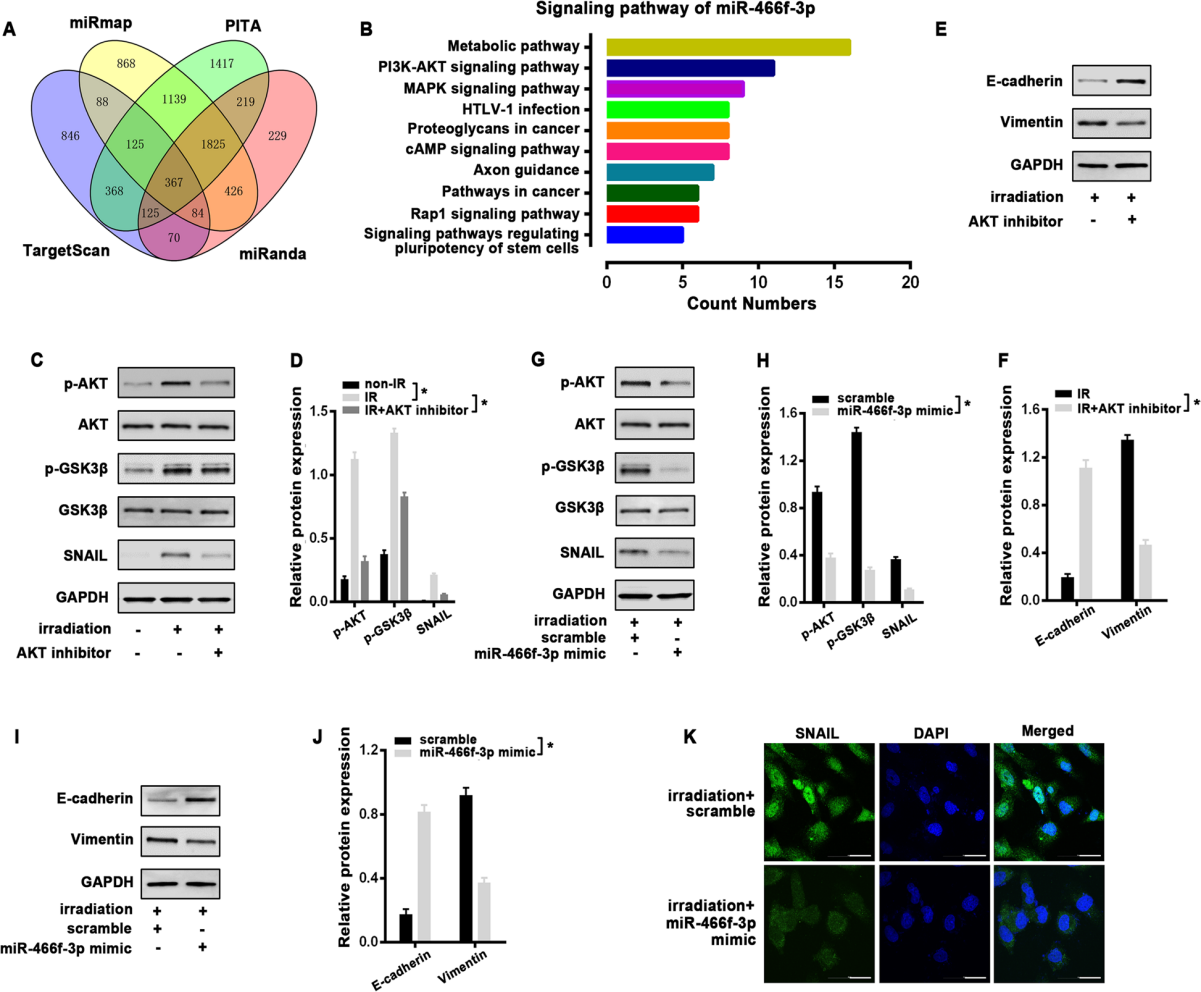
miR-466f-3p-dependent AKT/GSK3β pathway was critical for radiation-induced EMT in MLE-12 cells. **A** The overlap of potential targets of miR-466f-3p predicted by Targetscan, miRmap, PITA, and miRanda databases. **B** KEGG (Kyoto Encyclopedia of Genes and Genomes) pathway analysis for a panel of 367 overlapped targets of miR-466f-3p, and PI3K/AKT pathway was included among the top 10 enrichments. **C**, **D** Western blot analysis (C) and densitometric quantification (D) of p-AKT, p-GSK3β, and SNAIL in nonirradiated control and irradiated cells with or without AKT inhibitor (LY294002) at 1 h post-irradiation. **E**, **F** Western blot analysis (E) and densitometric quantification (F) of E-cadherin and Vimentin in irradiated cells with or without AKT inhibitor at 48 h post-irradiation. **G-J** Western blot analysis and densitometric quantification of p-AKT, p-GSK3β, and SNAIL (G, H), E-cadherin and Vimentin (I, J) in irradiated MLE-12 cells with scramble and miR-466f-3p mimic at 1 or 48 h post-irradiation. All the above western blot analysis used AKT, GSK3β, or GAPDH as the loading control. **K** Immunoflurescence staining for SNAIL (green) and DAPI (blue) in irradiated MLE-12 cells with scramble and miR-466f-3p mimic at 48 h post-irradiation. Data are presented as the mean ± SD. from three independent experiments, * *p* < 0.05

To better clarify the molecular mechanisms of miR-466f-3p-regulated EMT, the possible targets of miR-466f-3p were predicted on publicly available databases, including Targetscan, miRmap, PITA, and miRanda. A panel of 367 overlapped genes was subsequently annotated by Kyoto Encyclopedia of Genes and Genomes (KEGG) analysis (Fig. 5A). Interestingly, PI3K/AKT pathway (*KEGG* mmu04151) was one of the top 10 enrichments, which has been implicated in the induction of EMT (Fig. 5B). GSK3β, is a well-known kinase involved in many signaling pathways, whose activity depends on the phosphorylation site of amino acid. The inhibited GSK3β with phosphorylated serine-9 residue results in the upregulation of SNAIL, which is one of the important transcriptional repressors of E-cadherin, thus triggering EMT process [23, 24]. The previous references prompted us to investigate the response of AKT and GSK3β to radiation in MLE-12 cells firstly. Western blot analysis showed that AKT was obviously phosphorylated at Serine 473 (p-AKT) at 1 h after a single dose of 8 Gy radiation, accompanied with an increased phosphorylation of GSK3β at Serine 9 (p-GSK3β) and a corresponding increment of SNAIL (Fig. 5C, D). Then, we pretreated MLE-12 cells with the AKT inhibitor LY294002 for 2 h before radiation. When AKT signaling was blocked, radiation-induced repression of GSK-3β was released, which correlated with the downregulation of SNAIL (Fig. 5C, D). And AKT inhibitor also effectively reversed radiation-induced EMT by modulating the protein levels of E-cadherin and Vimentin at 48 h post-irradiation (Fig. 5E, F). Based on the above results, we examined the potential relationship between miR-466f-3p and AKT/GSK3β pathway. In Contrast to scrambled control, miR-466f-3p overexpression with its mimic in MLE-12 cells markedly decreased the strong signals of p-AKT and p-GSK3β following radiation, coupled with a reduction of Snail protein. Whereas the levels of total AKT and GSK3β were not affected (Fig. 5G, H). Furthermore, miR-466f-3p mimic obviously blunted radiation-induced EMT in MLE-12 cells by restoring E-cadherin expression, whereas it clearly inhibited the protein level of Vimentin (Fig. 5I, J). Moreover, immunofluorescence also showed that miR-466f-3p mimic concomitantly reversed the radiation-induced nuclear accumulation of Snail compared to scrambled control (Fig. 5K).

### C-MET, a direct target of miR-466f-3p, abrogates radiation-induced EMT via AKT/GSK3β pathway

**Fig. 6:**
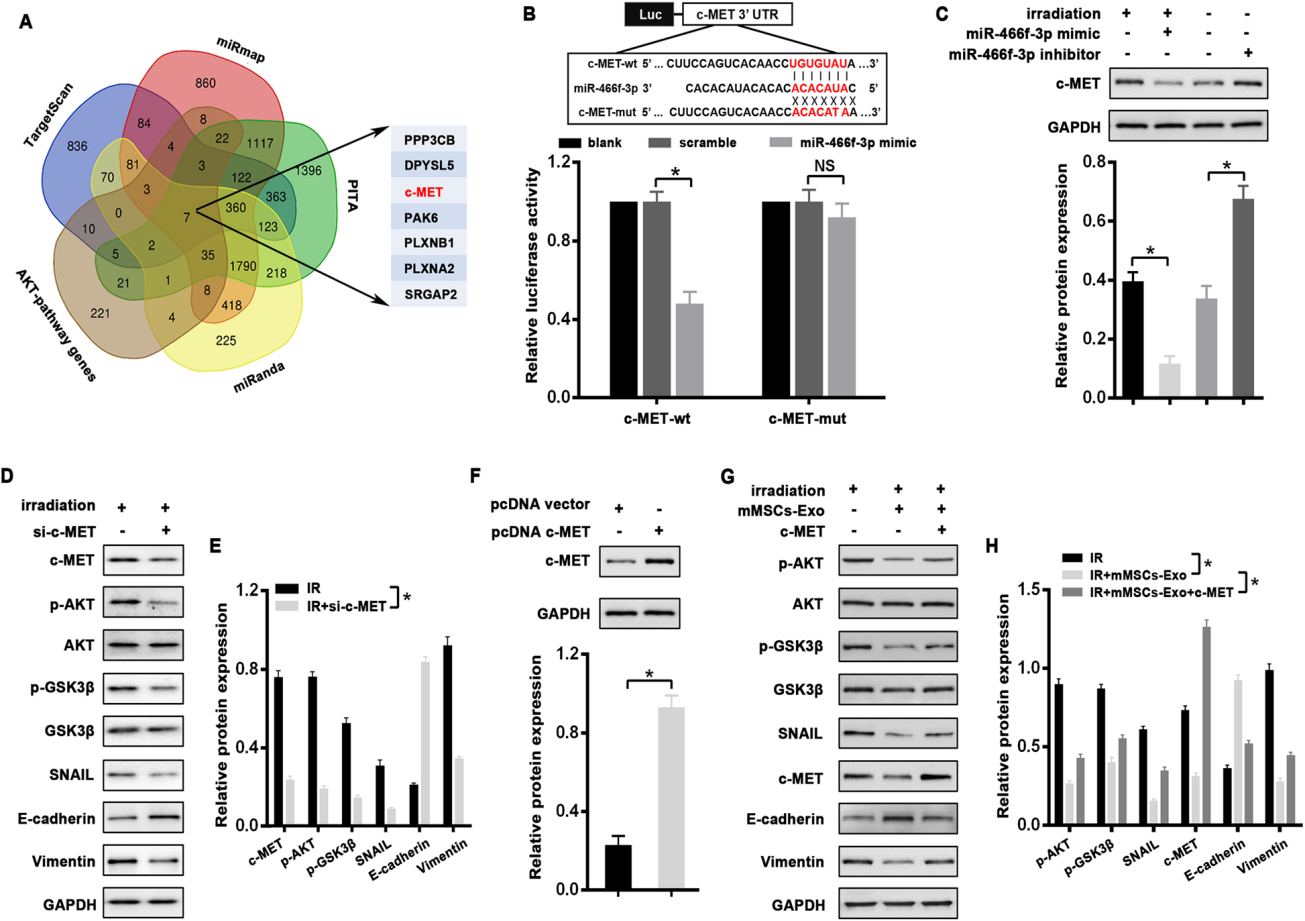
C-MET as a direct target of miR-466f-3p abrogated radiation-induced EMT via AKT/GSK3β pathway in MLE-12 cells. **A** Seven candidates overlapped by AKT-pathway genes and potential targets predicted by Targetscan, miRmap, PITA, and miRanda databases. **B** Schematic illustration of the potential binding sites for miR-466f-3p in the 3’-UTRs of c-MET (*upper*). Dual-luciferase assay in H293T cells after co-transfected either wild-type (c-MET-wt) or mutant (c-MET-mut) vectors with miR-466f-3p mimic *(lower)*. **C** Western blot and densitometric quantification for protein level of c-MET and GAPDH (for normalization) in irradiated MLE-12 cells with miR-466f-3p mimic and MLE-12 cells with miR-466f-3p inhibitor. **D**, **E** Western blot (D) and densitometric quantification (E) for protein level of c-MET, p-AKT, p-GSK3β, SNAIL, E-cadherin and Vimentin in irradiated MLE-12 cells with or without siRNA against c-MET. AKT, GSK3β, or GAPDH was used as the loading control. **F** Western blot and densitometric quantification for protein level of c-MET and GAPDH (for normalization) in c-MET expressing MLE-12 cells. **G**, **H** Western blot (G) and densitometric quantification (H) for protein level of p-AKT, p-GSK3β, SNAIL, c-MET, E-cadherin and Vimentin in irradiated MLE-12 cells with or without mMSCs-Exo treatment and c-MET restoration at 48 h post-irradiation. AKT, GSK3β, or GAPDH was used as the loading control. Data are presented as the mean ± SD. from three independent experiments, * *p* < 0.05

To verify the critical target of miR-466f-3p responsible for AKT inhibition, the aforementioned predicted genes were overlapped with the *KEGG* PI3K-AKT pathway. Among the candidates, we focused on c-MET (Fig. 6A), whose role in lung fibrosis has been documented. Firstly, the direct interaction between miR-466f-3p and c-MET was testified by luciferase assay. We found that miR-466f-3p mimic significantly suppressed the luciferase activity of the reporter gene with wild-type 3’UTR of *c-MET,* whereas this inhibitory effect was markedly abolished when the vector containing *c-MET-*mutated 3’UTR (Fig. 6B). Additionally, as compared to NC control, transfection of miR-466f-3p mimic significantly decreased c-MET protein in irradiated MLE-12 cells, whereas c-MET expression was increased in miR-466f-3p-inhibiting MLE-12 cells (Fig. 6C). Moreover, knockdown c-MET via siRNA markedly inhibited radiation-induced phosphorylation of AKT and GSK3β, and thus reversed the EMT phenotype in irradiated MLE-12 cells (Fig. 6D, E). To further support the role of c-MET in mMSCs-Exos-mediated EMT process, an expression vector lacking 3’UTR of c-MET (pcDNA c-MET) was transfected into MLE-12 cells, and its ectopic expression was identified as compared to the control (Fig. 6F). As expected, overexpressing c-MET attenuated the radiation-induced changes in MLE-12 cells treated with mMSCs-Exos, including p-AKT, p-GSK3β, SNAIL, and EMT markers E-cadherin and Vimentin (Fig. 6G, H).

## Discussion

Radiation-induced lung fibrosis is a serious and life-threatening complication of thoracic radiotherapy. With the prolonged survival of cancer patients, the concerns for long-term complications of radiotherapy are increasing. However, current treatments for RILF have not significantly improved survival, thus new approaches have been critically urgent in clinic. In the present study, the protective role of mMSCs-Exos was identified in a mouse model of radiation-induced lung injury, which also protected alveolar epithelial cells from radiation-induced EMT process. Moreover, we demonstrated miR-466f-3p in mMSCs-Exos as a key component to reverse radiation-induced EMT through c-MET/AKT/GSK3β pathway. To a certain extent, our findings provide new insights into the potential prevention of the fibrotic events following radiation injury.

MSCs, as a population of multipotent cells, are considered to be a potential source for regeneration of damaged tissues [25, 26]. However, many hurdles exist for translating the therapeutic promise of MSCs from bench to bed [27]. As compared to cell therapy, exosomes have several advantages for clinical application, which are more stable in tissues and body fluids and exhibit low levels of immunogenicity and toxicity [28]. Thus, exosomes may serve as a promising alternative with properties similar to their parental cells, and indeed, MSCs-derived exosomes have therapeutic effects on lung injury and fibrosis. A recent paper showed that MSCs-derived exosomes efficiently attenuated hypoxia-induced lung injury, whereas exosome depletion totally abolished these protective effects. Herein, we extended the biologic effect of mMSCs-derived exosomes in mice model with radiation-induced lung injury. Our *in-vivo* data showed that mMSCs-Exos treatment obviously reduced alveolar inflammation, inhibited collagen deposition and maintained better histologic architecture in lung tissues injured by radiation, strongly suggesting that MSCs-derived exosomes may represent an ideal cell-free modality to mitigate radiation-induced lung injury. However, clinical application of exosomes therapy has been fraught with several technical challenges to identify, isolate and quantify exosomes accurately and efficiently.

Emerging evidence has revealed that injured alveolar epithelial cells suffering EMT process are main sources of myofibroblasts generation [29]. In line with previous studies [8, 30], the present work consistently confirmed EMT event induced by radiation in murine alveolar epithelial cells MLE-12, characterized by a loss of epithelial polarity, downregulation of epithelial marker E-cadherin expression and conversely upregulation of mesenchymal marker Vimentin. Intriguingly, we demonstrated that MSCs-derived exosomes transmitted horizontally to MLE-12 cells efficiently suppressed the radiation-induced EMT process. Recently, miRNAs cargo in MSCs exosomes have gained increased recognition for their important role in tissue repair and regeneration by interfering with gene expression in recipient cells [31, 32]. miRNAs are selectively packaged into exosomes depending on the animal type and parental cell source [33]. Based on microarray assay, we showed that mMSCs-Exos administration greatly upregulated miR-466f-3p in radiated MLE-12 cells, which was highly enriched in mMSCs-Exos. miR-466f-3p has been shown to be a potential target for cerebral ischemia [34]. Another study reported that low expression of miR-466f-3p promoted more mesenchymal phenotype in medulloblastoma stem cells through Vegfa-Nrp2 signaling pathway [35]. However, the function of miR-466f-3p in lung injury is scant and scarce. Herein, we first demonstrated that knocking down miR-466f-3p in mMSCs-Exos obviously attenuated their function in the EMT inhibition, as evidenced by maintaining more mesenchymal phenotypes in injured cells, which supported that miR-466f-3p successfully transferred into injured MLE-12 cells through exosomal delivery was mainly responsible for the protective role of mMSCs-Exos. However, further studies are warranted to determine whether other materials presenting in exosomes are involved in this process. With the in vivo application of genetically modified MSCs in fibrotic disease [36], MSCs exosomes engineered to overexpress miR-466f-3p is likely to be a more efficient strategy for the enhanced ability of MSCs to treat radiation-induced lung injury.

Besides the classical Wnt/β-catenin and TGF-β signaling pathways [6, 37], the aberrant activation of PI3K/AKT pathway has also been proven vitally in EMT induction and tissue fibrogenesis [38, 39]. To data, constitutive activity of GSK-3β is a vital signaling mediator that can facilitate the epithelial architecture via a reverse of EMT process, thereby exerting its protective role against the tissue fibrosis [40, 41]. Whereas inhibited GSK-3β suppresses the phosphorylation of SNAIL and induces the nuclear localization of SNAIL [42], which subsequently binds to the E-boxes of the E-cadherin promoter and represses its expression [23]. Recent studies showed that GSK-3β inhibitor upregulated p-GSK3β at serine-9 residue and SNAIL expression, confirming that GSK-3β inhibition is involved in stabilization of SNAIL [43]. Herein, we found that radiation activated AKT and suppressed GSK-3β activity. With the help of an AKT inhibitor, we identified that AKT was upstream of GSK-3β and the inactivation of GSK3β via AKT paralleled the increase of SNAIL. In addition, ectopic expression of miR-466f-3p prevented the radiation-induced modulation of AKT, GSK3β, SNAIL, E-cadherin and Vimentin protein levels in MLE-12 cells. Altogether, our data implied that miR-466f-3p-mediated-AKT/GSK-3β pathway is required for radiation-induced EMT in lung alveolar epithelial cells. Nevertheless, the molecular basis how miR-466f-3p downregulated AKT has been still obscure. So we overlapped the predicted targets of miR-466f-3p with the genes involved in *KEGG* PI3K-AKT pathway. With the help of luciferase reporter assay and western blot analysis, c-MET was identified as a crucial novel target of miR-466f-3p. *C-MET* gene, encoding for the tyrosine kinases receptor for hepatocyte growth factor (HGF), could collaborate in maintaining tissue plasticity and the regenerative potential that characterizes pulmonary fibrosis [44, 45]. Aberrant c-MET signaling, a frequent cause of direct phosphorylation and activation of AKT [46], could be post-transcriptionally regulated via miRNAs. Zhang *et al.* showed that miR-338-3p inhibited cells growth and metastasis of ovarian cancer by reversing EMT via targeting the c-MET gene [47]. And the research by *Xu et al.* also demonstrated that miR-433 could inhibit invasive phenotype of bladder cancer via targeting c-MET [48]. Consistently with the reports in cancers, we verified that upregulation of miR-466f-3p is a new mechanism responsible for c-Met inhibition. And we further showed that ectopic expression of c-MET promoted the activating phosphorylation of AKT and the inhibitory phosphorylation of GSK3β, thus attenuated the EMT-inhibition induced by miR-466f-3p-enriched mMSCs-Exos. All these data support the notion that exosomal miR-466f-3p regulates radiation-induced EMT via c-Met/AKT/GSK3β pathway. Given that various miRNAs could have the same or different target genes, the complex regulatory network of miRNAs await further validation in future

## Conclusions

**Fig. 7:**
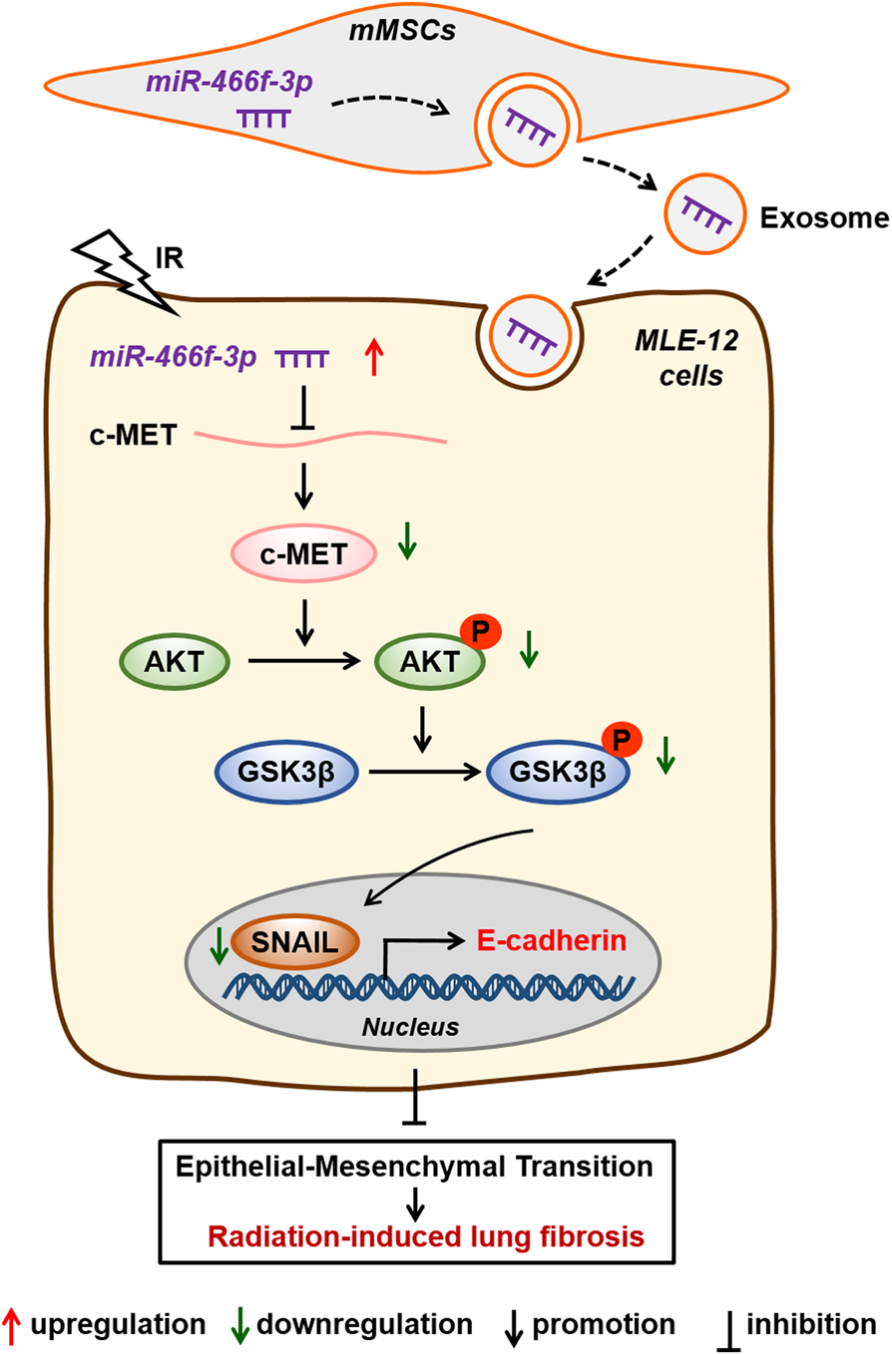
The schematic cartoon illustrated how mMSCs-derived exosomal miR-466f-3p protected the mouse pulmonary from radiation-induced injury through AKT/ GSK3β pathway by targeting *c-MET*

In summary, we demonstrated that mMSCs-Exos alleviated radiation-induced lung injury in vivo and in vitro. In particular, exosomal miR-466f-3p transferred from mMSCs into radiation-injured MLE-12 cells targeted c-MET, thereby downregulation of AKT/GSK3β signaling pathway, thus inhibited radiation-induced EMT event (Fig. 7). Most importantly, this understanding of mMSCs-Exos in radiation-induced lung injury may improve our prospects for developing preventive approaches.

## Supplementary Information

**Additional file 1: Table S1.** RT-PCR Primers used in this study.

## Data Availability

Not applicable.

## Abbreviations

MSC: Mesenchymal stem cells
EMT: Epithelial-mesenchymal transition
mMSCs-Exos: Mouse mesenchymal stem cells-derived exosomes
IR: Ionizing radiation
RILF: Radiation-induced lung fibrosis
RILI: Radiation-induced lung injury
miRNA: MicroRNA
