# Supplementary material for: Mouse mesenchymal stem cell-derived exosomal miR-466f-3p reverses EMT process through inhibiting AKT/GSK3β pathway via c-MET in radiation-induced lung injury
Source: J Exp Clin Cancer Res. 2022 Apr 7;41:128. doi: 10.1186/s13046-022-02351-z (PMC8988379; doi:10.1186/s13046-022-02351-z)
Supplement: Supplementary file 1 — Additional file 1: Table S1. RT-PCR Primers used in this study. [file 13046_2022_2351_MOESM1_ESM.docx]

**Table S1. RT-PCR Primers used in this study**

| **Gene symbol** | **Sequence (5’-3’)** |
| --- | --- |
| mmu-miR-30f | Forward: 5’-CGGCGTAAACATCCGACTGA-3’ |
| mmu-miR-1a-3p | Forward: 5’-CCGGCTGGAATGTAAAGAAGT -3’ |
| mmu-miR-203-5p | Forward: 5’-CGGCAGTGGTTCTTGACAGT-3’ |
| mmu-miR-212-5p | Forward: 5’-CGGCACCTTGGCTCTAGACTG -3’ |
| mmu-miR-341-3p | Forward: 5’-GCTCGGTCGATCGGTCG-3’ |
| mmu-miR-543-3p | Forward: 5’-GCAAACATTCGCGGTGCA-3’ |
| mmu-miR-504-5p | Forward: 5’-GGCAGACCCTGGTCTGCAC-3’ |
| mmu-miR-133a-5p | Forward: 5’-CGGCGCTGGTAAAATGGAA-3’ |
| mmu-miR-466i-3p | Forward: 5’-GCCGGCATACACACACACATAC-3’ |
| mmu-miR-466f-3p | Forward: 5’-CCGGCCATACACACACACAT-3’ |
| common | Reverse: 5’-ACTGCAGGGTCCGAGGTATT-3’ |
| reverse transcription | 5’-GTCGTATCGACTGCAGGGTCCGAGGTATTCGCAGTCGATACGAC-3’ |
